# Metabolic and Behavioral Compensations in Response to Caloric Restriction: Implications for the Maintenance of Weight Loss

**DOI:** 10.1371/journal.pone.0004377

**Published:** 2009-02-09

**Authors:** Leanne M. Redman, Leonie K. Heilbronn, Corby K. Martin, Lilian de Jonge, Donald A. Williamson, James P. Delany, Eric Ravussin, for the Pennington CALERIE team

**Affiliations:** Pennington Biomedical Research Center, Baton Rouge, Louisiana, United States of America; University of Louisville, United States of America

## Abstract

**Background:**

Metabolic and behavioral adaptations to caloric restriction (CR) in free-living conditions have not yet been objectively measured.

**Methodology and Principal Findings:**

Forty-eight (36.8±1.0 y), overweight (BMI 27.8±0.7 kg/m^2^) participants were randomized to four groups for 6-months; *Control*: energy intake at 100% of energy requirements; *CR*: 25% calorie restriction; *CR+EX*: 12.5% CR plus 12.5% increase in energy expenditure by structured exercise; *LCD*: low calorie diet (890 kcal/d) until 15% weight reduction followed by weight maintenance. Body composition (DXA) and total daily energy expenditure (TDEE) over 14-days by doubly labeled water (DLW) and activity related energy activity (AREE) were measured after 3 (M3) and 6 (M6) months of intervention. Weight changes at M6 were −1.0±1.1% (Control), −10.4±0.9% (CR), −10.0±0.8% (CR+EX) and −13.9±0.8% (LCD). At M3, absolute TDEE was significantly reduced in CR (−454±76 kcal/d) and LCD (−633±66 kcal/d) but not in CR+EX or controls. At M6 the reduction in TDEE remained lower than baseline in CR (−316±118 kcal/d) and LCD (−389±124 kcal/d) but reached significance only when CR and LCD were combined (−351±83 kcal/d). In response to caloric restriction (CR/LCD combined), TDEE adjusted for body composition, was significantly lower by −431±51 and −240±83 kcal/d at M3 and M6, respectively, indicating a metabolic adaptation. Likewise, physical activity (TDEE adjusted for sleeping metabolic rate) was significantly reduced from baseline at both time points. For control and CR+EX, adjusted TDEE (body composition or sleeping metabolic rate) was not changed at either M3 or M6.

**Conclusions:**

For the first time we show that in free-living conditions, CR results in a metabolic adaptation and a behavioral adaptation with decreased physical activity levels. These data also suggest potential mechanisms by which CR causes large inter-individual variability in the rates of weight loss and how exercise may influence weight loss and weight loss maintenance.

**Trial Registration:**

ClinicalTrials.gov NCT00099151

## Introduction

Daily energy expenditure has three major components: resting metabolic rate, the thermic effect of food and the energy cost of physical activity. Respiratory chambers enable the measurement of sleeping metabolic rate, the energy cost of arousal, the thermic effect of food and the energy cost of spontaneous physical activity [1]. However the confined space within a respiratory chamber compromises the level of habitual and/or voluntary physical activity. Activity thermogenesis is the most variable component of daily energy expenditure and includes energy expended during both voluntary physical activity and all other non-exercise activities [2]. The latter, recently termed NEAT (non-exercise activity thermogenesis) includes the energy cost of sitting, fidgeting, maintaining posture, muscle tone and leisure activities such as playing guitar and shopping etc [3].

Caloric restriction (CR) is the most robust non-pharmacological intervention known to increase maximum lifespan. One of the most popular proposed theories by which CR promotes lifespan extension is the rate of living theory [4]. We hypothesized that caloric restriction will reduce energy metabolism in excess of the loss of metabolic mass (metabolic adaption) and therefore will reduce oxidative damage to tissues and organs by lowering the production rates of reactive oxygen species [5]. CR is indeed associated with robust decreases in energy metabolism, including a lowering of resting metabolic rate (or sleeping metabolic rate), reduced thermic effect of meals and a decrease in the energy cost and/or the level of physical activity. However, it is debated whether or not the decrease in total energy expenditure is proportional (or larger; metabolic adaptation) to the loss of metabolic tissues (fat-free and fat mass: FFM and FM). Furthermore, it is not clear if the metabolic adaptation persists once a new stable body weight is reached. Previously we reported that both 24-hour sedentary energy expenditure and sleeping metabolic rate measured in a respiratory chamber were reduced ∼6% beyond what was expected for the loss of metabolic mass (FFM and FM) [6]. This metabolic adaptation was also observed in RMR measured by a ventilated hood indirect calorimeter [7]. A portion of the reduction in sedentary energy expenditure was due to the reduced energy intake itself (thermic effect of food), and a reduction in the energy cost of spontaneous physical activity. The majority however, was due to the decline in the size of the metabolizing mass and a lowering of the rate of metabolism per mass unit of tissues and organs.

Essential to the study of the changes in energy expenditure with CR is an objective assessment of physical activity. Not only is the contribution of physical activity to daily energy expenditure quite variable, it is also likely that in an attempt to conserve energy during CR, individuals volitionally or non-volitionally decrease their level of physical activity [8]. In our study of 25% CR in overweight humans, we observed no change in spontaneous physical activity in a respiratory chamber [7] consistent with earlier reports of no alterations in spontaneous physical activity [9] or posture allocation in obese individuals following weight loss [10]. These findings are not surprising if the current hypothesis that spontaneous physical activity is biologically determined and not altered by perturbations in body weight, is true [2], [10].

Combining the doubly labeled water method and indirect calorimetry allows us to disentangle sedentary metabolic adaptation from behavioral responses in physical activity. The objective of the current study therefore was to determine if CR induces a reduction in free-living energy expenditure that is larger than what can be explained by changes in body weight and body composition (i.e. metabolic adaptation). Furthermore we wished to examine whether CR induced a change in energy expended in physical activity [6]. As a secondary analysis we determined if metabolic adaptation occurred only during weight loss or if it persisted after the lower body mass reached stability. We hypothesized that similar to sedentary energy expenditure, total daily energy expenditure would be reduced by CR partly due to metabolic adaptation and partly due to decreased physical activity. Such metabolic and behavioral adaptation can explain the variability in the rates of weight loss even in well controlled studies as well as the propensity of relapse after weight loss.

## Methods

### Ethics Statement

This study was conducted according to the principles expressed in the declaration of Helsinki. The study was approved by the Pennington Biomedical Research Center IRB and the Data Safety Monitoring Board of CALERIE. All participants provided written informed consent for the collection of samples and subsequent analysis. The protocol for this trial and supporting CONSORT checklist are available as supporting information; see Checklist S1 and Protocol S1.

### Participants

**Figure 1:**
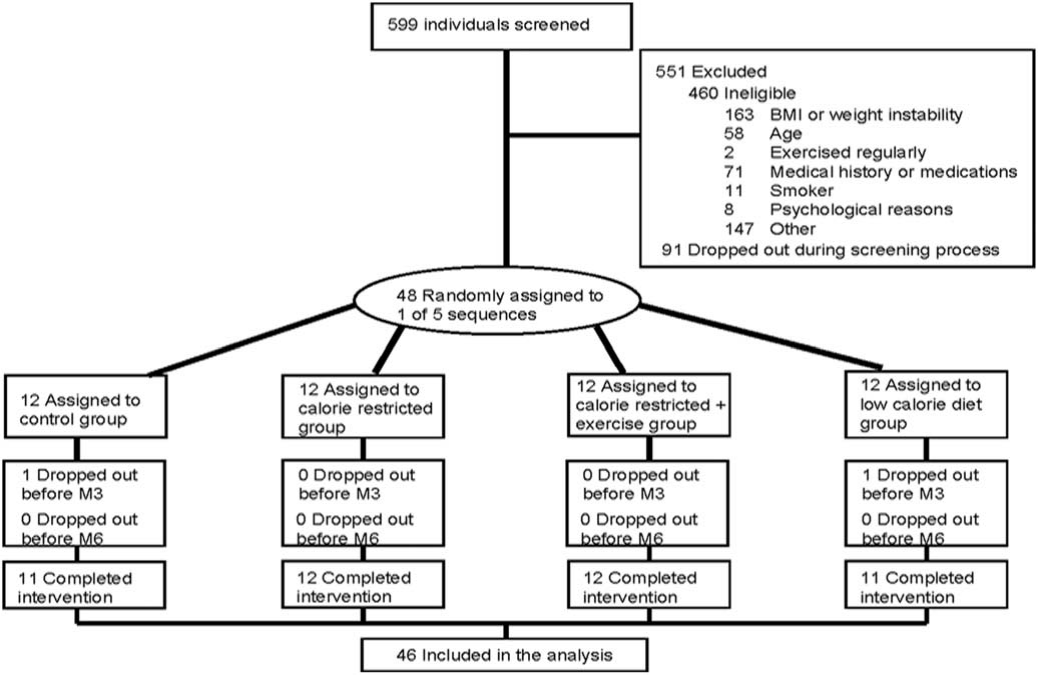
Flow of participants through the Pennington Phase 1 CALERIE trial. The CONSORT diagram was published previously [6].

Of the 599 individuals screened for the study, 551 were excluded (460 were ineligible; 91 withdrew during screening) (Figure 1). Forty-eight healthy, overweight (25≥BMI<30) men (25–50 y) and women (25–45 y) were enrolled in the study. The physical characteristics of the participants during weight maintenance at baseline are summarized in Table 1. Participants were excluded if they smoked, exercised more than twice a week, were pregnant, lactating or post-menopausal, had a history of obesity (BMI>32), diabetes, cardiovascular disease, eating disorders, psychological disorders, substance abuse or regularly used medications except for birth control. Details of the screening process and study population have been previously described [6].

**Table 1:** Physical characteristics of 48 men and women in weight maintenance at baseline.

	Male (n = 21)		Female (n = 27)	
Age, *y*	38±7	[27–50]	38±6	[27–45]
Weight, *kg*	89.2±9.0	[77–105]	76.1±7.0	[61–92]
BMI, *kg·m^2^*	27.9±1.7	[26]–[30]	27.7±1.8	[25]–[30]
Body Fat, *%*	24.8±3.1	[17]–[31]	37.6±4.1	[29–45]
Fat mass, *kg*	22.1±3.8	[14]–[29]	28.8±5.1	[19]–[38]
Fat-free mass, *kg*	67.1±6.8	[56–79]	47.3±3.6	[39–58]

Data are mean±SE and [range].

### Study design

**Figure 2:**
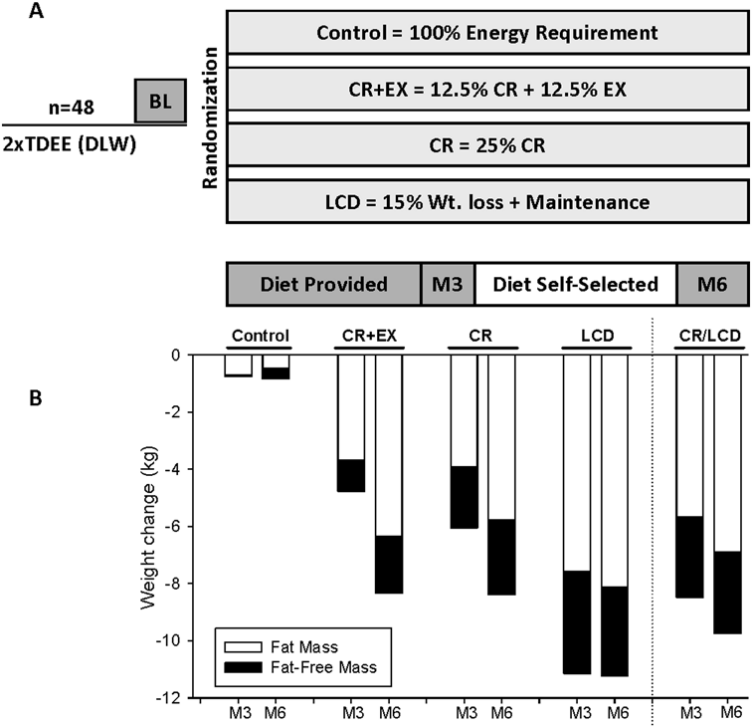
Experimental design (A) and body weight and composition changes (B) at the completion of the study.

In brief, participants were randomized into one of four groups for 24 weeks: control = healthy weight maintenance based on an American Heart Association (AHA) Step 1 diet, CR = 25% caloric restriction from baseline energy requirements, CR+EX = 12.5% caloric restriction and 12.5% increase in energy expenditure through structured aerobic exercise and LCD = low calorie diet (890 kcal/d) to achieve a 15% reduction in body mass followed by weight maintenance (Figure 2). The group assignment was stratified to ensure equal distributions of sex and BMI in the four groups. All physiological and psychological testing was conducted at baseline and at the end of weeks 12 (M3) and 24 (M6) over a 5-day stay in the institutional inpatient unit. Where possible study personnel were blinded to the treatment assignment of the subjects.

### Baseline weight-maintenance energy requirements

The energy intake required for weight maintenance during baseline and the subsequent energy deficit prescribed to achieve the desired caloric restriction were calculated from 4-week data including two 14-day periods by doubly labeled water (DLW). During the first DLW study (B1) participants followed their usual diet at home. During the second DLW study (B2), participants were provided with a weight maintenance diet. During B2, subjects were weighed every morning and changes in body weight were obtained by regression analysis every 3 days over the 2 week period and energy intake was adjusted as needed to maintain a stable body weight (<±250 g). Values from the animal literature for tissue gain or loss were used to assign energy values to weight changes as previously described [11] and adjustments were made to determine energy intake for weight maintenance. Individual energy requirements were then calculated as the average of total daily energy expenditure (TDEE) during B1 and the final energy intake for weight maintenance (after adjustments) during B2.

### Diet and behavioral intervention

During weeks 1–12 and 22–24 of the intervention, participants were provided with all meals prepared by the metabolic kitchen at the Center and based on individual energy intake targets. On weekdays, breakfast and dinner were eaten at the center. All lunches, snacks and weekend meals were packaged for take-out. During weeks 13–22 participants self-selected a diet based on their individual targets. The diet composition was based on the AHA guidelines, 30% calories from fat, 15% from protein and 55% from carbohydrate. During the self-selected feeding, compliance to the prescribed dietary intervention was monitored from self-reported food records and changes in body weight which were reviewed weekly during behavioral group or individual sessions. During these weekly meetings, participants learned cognitive-behavioral techniques on how to adhere to their meal and exercise plans.

### Exercise prescription and compliance

Participants in CR, LCD and Control groups were required to continue their usual pattern of physical activity while participants in CR+EX increased their energy expenditure by 12.5% above baseline through structured aerobic exercise, 5 days per week. At least three of the five weekly exercise sessions were performed under supervision at the exercise facility of our center and heart rate monitor data was reviewed for compliance for all session conducted outside the center. The exercise program was implemented gradually and by week 6 all participants were expending the required 12.5% of baseline energy expenditure. Exercise energy expenditure was initially determined by indirect calorimetry and thereafter HR was used to assess compliance during all exercise sessions. The target energy cost was maintained at 403±63 kcal per session for women and 569±118 kcal per session for men throughout the entire intervention resulting in average exercise durations of 53±11 and 45±14 min per session for women and men, respectively.

### Doubly labeled water

Besides the two baseline measurements, total daily energy expenditure (TDEE) was measured by 14-day doubly labeled water during weeks 10–12 (M3) and 22–24 (M6) of the intervention. Briefly, subjects provided two urine samples before being dosed with labeled water (2.0 g of 10% enriched H_2_^18^O and 0.12 g of 99.9% enriched ^2^H_2_O per kg of estimated total body water from DXA) and 2 samples at 4.5 and 6h after dosing. Urine samples collected at 1.5 and 3 hours were discarded. On days 7 and 14 after dosing, subjects provided two additional timed urine samples. Each sample was analyzed for ^18^O and ^2^H abundance by isotope ratio mass spectrometry [7]. The isotopic enrichments of the post-dose urines compared with the pre-dose samples were used to calculate elimination rates (k_H_ and k_O_) using linear regression and initial isotope dilution spaces were calculated by extrapolation to time 0. CO_2_ production rate (rCO_2_) was calculated using the equations of Schoeller et al. [14] as modified by Racette et al [15]. Total energy expenditure was calculated by multiplying rCO_2_ by the energy equivalent of CO_2_ for an RQ of 0.860 at B1 corresponding to 30% fat; 55% CHO and 15% protein while for B2 the energy equivalent of CO_2_ was based on the calculated food quotient of the diet (FQ = 0.882). For months 3 and 6, during the intervention when weight loss was observed, energy expenditure was calculated from rCO2 by using a metabolic fuel quotient derived from food intake, changes in body energy stores and conventional calorimetric relations corrected for the changes in fat mass (FM) and fat free mass (FFM) as previously described [11], [16].

### Body composition

Metabolic weight was determined by the mean of two consecutive measurements obtained in the morning following a 12 h fast and morning void and corrected for the weight of a hospital gown. Whole body percent body fat was measured using DXA (Hologics QDR 4500A, Bedford, MA) and FM and FFM were calculated from the percent body fat and the metabolic body weight.

### Sedentary energy expenditure

Sedentary EE (24h-EE) was measured over 23 hours in a whole room indirect calorimeter as previously described [6], [17]. In keeping with the assigned interventions, at M3 and M6 controls were fed the same number of calories on their return visits to the metabolic chamber whereas CR subjects were fed 25% less and CR+EX subjects 12.5% less than measured baseline 24h-EE. At M3, LCD participants were fed so that energy intake was tightly matched to measured energy expenditure and provided the same meals at M6. Sleeping metabolic rate (SMR) was calculated between 02:00–05:00 am, when no motion was detected.

### Physical activity

Physical activity was estimated from daily energy expenditure by doubly labeled water and sleeping metabolic rate using two different calculations. First the physical activity level (PAL) was calculated by the widely accepted method of dividing TDEE by SMR (PAL = TDEE/SMR). Because of the inherent problem of using ratios when the two variables have an intercept not equal to zero [18], we also expressed physical activity as the residual value of the regression between measured TDEE and measured SMR [19]. This value we termed Activity Related Energy Expenditure (AREE), is positive for subjects with higher physical activity than average and negative for subjects with lower physical activity than average independent of metabolic body size. Because AREE is adjusted for metabolic body size (SMR), this value is directly proportional to the amount of physical activity.

### Psychological testing

Psychological testing included an assessment of health related quality of life. The Medical Outcomes Study Short-Form 36 Health Survey (SF-36) is a self-administered 36-item questionnaire that measures health related quality of life. The validity and reliability of the SF-36 have been established [12], [13]. In the present study, the SF-36 was used to measure vitality (VT) and physical functioning (PF). Participants' raw scores were converted into scale scores ranging from 0 to 100, with higher scores representing better QOL or higher levels of functioning.

### Statistical analysis

Data in the text and tables are provided as means±SE. SAS Version 9.12 (SAS Institute, Cary, NC) was used for analysis. The change in variables from baseline to M3 and M6 and M3 and M6 were analyzed by repeated measures with treatment and time interactions and baseline values included as covariates. Five groups were used in the analysis including the 4 study intervention groups (CR, CR+EX, LCD and Control) and a group combining both CR and LCD (n = 23). To control for type I error, statistical significance for all multiple comparisons was adjusted using the Tukey-Kramer method. Multiple linear regression models at baseline (n = 48) were used to generate equations for predicting TDEE. Using the latter equation, predicted values for TDEE were generated at M3 and M6 using the equation with the actual measured FFM and FM, or actual weight or SMR. The partial coefficients for each model are reported and the differences between the measured and predicted TDEE values were calculated and analyzed using ANOVA. *P*<0.05 was considered statistically significant.

## Results

### Total daily energy expenditure at baseline

At baseline TDEE was not different between the four treatment groups (Table 2). Baseline TDEE correlated with FFM (r = 0.85, *P*<0.001), weight (r = 0.71, *P*<0.0001) and FM (r = 0.38, *P*<0.01).

**Table 2:** Total daily energy expenditure (TDEE; kcal/day) measured by doubly labeled water at M0, M3 and M6.

	TDEE	Change in TDEE not explained by FFM and FM	*P * *
**Control**			
-BL	2879±148	-	-
-M3	2753±144	−134±91	*NS*
-M6	2940±184	−13±185	*NS*
**CR+EX**			
-BL	2653±148	-	-
-M3	2603±146	−2±115	*NS*
-M6	2686±182	129±86	*NS*
**CR**			
-BL	2842±170	-	-
-M3	2388±148*	−371±75	*<0.0001*
-M6	2531±127*	−209±114	*NS*
**LCD**			
-BL	2812±135	-	-
-M3	2179±175*	−496±68	*<0.0001*
-M6	2373±128*	−275±127	*0.07*
**CR/LCD**			
-BL	2828±107	-	-
-M3	2288±114*	−431±51	*<0.0001*
-M6	2456±90*	−240±83	*0.01*

Predicted values were calculated on the basis of the equations generated at baseline. A significant *P* value indicates “metabolic adaptation”, i.e. the measured value is below the predicted value for the new metabolic mass (fat-free mass and fat mass). Data are mean±SE for the 46 participants who completed the study.

* indicates a significant change from baseline.

The prediction equation for relating TDEE to:

- FFM, FM, sex and age was: TDEE (kcal/d) = TDEE = 1630+33.4(FFM in kg) +1.9(FM in kg)−16.9(age in yr)−173(for females), r^2^ = 0.78;
- Weight, sex and age was: TDEE (kcal/d) = 2189 +19.6(weight in kg)−17.6(age in yr)−555(for females), r^2^ = 0.75;
- 24h-EE, sex and age was: TDEE (kcal/d) = 1551+0.95(24h-EE)−15.0(age in yr)−312(for females), r^2^ = 0.79;
- SMR, sex and age was: TDEE (kcal/d) = 2446+ 0.79(SMR)−16.7(age in yr)−511(for females), r^2^ = 0.73.

### Body weight and composition changes

At M3, body weight, FM and FFM were reduced from baseline in all three intervention groups and remained stable in the control group. By design, body weight and composition were stabilized in the LCD group between M3 and M6, while both continued to decrease in CR and CR+EX (Figure 2). At the completion of the 24-week study, body weight was reduced by 0.8±0.8 (−1.0±1.1%), 8.3±0.8 (−10.4±0.9%), 8.4±0.8 (−10.0±0.8%) and 11.2±0.6 kg (−13.9±0.8%) in control, CR, CR+EX and LCD, respectively (Figure 2B).

### Effect of CR on absolute TDEE

TDEE was not significantly changed in control subjects or in subjects assigned to CR+EX at M3 and M6 (Table 2). TDEE was lower during weight loss (Table 2) with 25%CR and during both the active weight loss phase (M3) and tended to be lower at weight loss maintenance (M6) in the LCD group (*p* = 0.07). For CR and LCD, TDEE at M6 (CR: −316±118; LCD: −389±124, kcal/d) was increased from M3 but still significantly lower than the baseline values and different from the control group. There was no difference in the change in TDEE from baseline at M3 and M6 in either CR or LCD.

### Effect of CR on TDEE adjusted for body composition

To determine if there was a metabolic adaptation to the CR intervention at M3 and M6 we compared the actual TDEE measured by DLW at each time point with the TDEE predicted from FFM and FM derived from the prediction equations generated at baseline and presented above. After adjustment for body composition (FFM and FM, Table 2) no metabolic adaptation was observed in control or CR+EX. With caloric restriction (CR/LCD combined), measured TDEE was significantly lower than TDEE predicted from body composition at M3 and M6. Furthermore, these “residuals” representing a metabolic adaptation were significantly lower than those in the control and CR+EX groups at both time points. Analyzed separately, however, actual TDEE was significantly lower than predicted values only at M3 (CR: −371±75; LCD: −496±68, kcal/d). At M6, the differential between the observed and predicted values for TDEE remained negative however did not reach statistical significance for either group (Table 2). Similar results were obtained when TDEE values were adjusted for weight loss instead of FFM and FM losses.

### Effect of CR on physical activity

**Figure 3:**
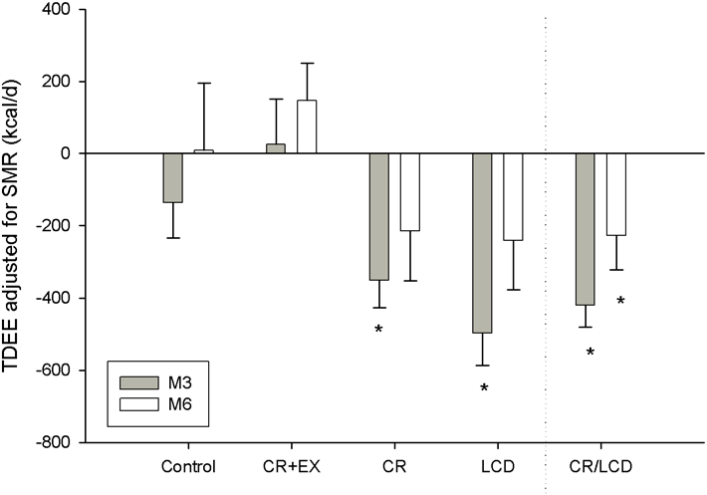
The effect of caloric restriction on AREE (change in TDEE at M3 and M6 after adjusting for SMR (a measure of sedentary energy expenditure). * represents a significant change from baseline.

We next adjusted TDEE for sedentary energy expenditure measured in a respiratory chamber (24h-EE) or for SMR. This adjustment allows us to disentangle the effect of physical activity from the effect of sedentary metabolism in response to CR. With both CR and LCD combined (CR/LCD, n = 23) TDEE adjusted for SMR and expressed as “residuals” (termed AREE, Figure 3) was significantly decreased (and different from controls) at both M3 (−420±60 kcal/d) and M6 (−228±93 kcal/d). No changes from baseline were observed in CR+EX or Control. This indicates a metabolic adaptation unrelated to sedentary energy expenditure and therefore resulting from decreased habitual or voluntary physical activity. When analyzed independently (Figure 3), after adjustment for SMR, TDEE (AREE) was significantly lower than TDEE predicted in CR (−350±76 kcal/d) and LCD (−497±90 kcal/d) at M3 but not at M6 (CR: −215±136, LCD: −241±135 kcal/d, *NS*). Consistently, there was a significant reduction in PAL (TDEE/SMR) at M3 in response to CR (CR/LCD combined). PAL and AREE were significantly lower at M3 than at baseline in LCD and CR but not in CR+EX or control. The reduction in PAL was no longer evident with further weight loss at M6 in CR or weight loss maintenance in LCD.

### Effect of CR on Physical Functioning and Vitality

According to the SF-36 survey, all treatment groups, but not the control group, reported improvement in physical functioning with the intervention. For CR, physical functioning was significantly improved from baseline (*p*<0.05 for all) at M3 (4±4%) and M6 (18±12%), whereas for CR+EX (3±1%) and LCD (1±1%) there was only a slight improvement at M6. The CR and LCD groups had significantly improved physical functioning compared to the control group at M3 (*p*<0.04) and M6 (*p*<0.04), and the CR+EX group had significantly improved physical functioning compared to control at M6 (*p* = 0.02). Vitality was not different from baseline in any treatment group and either time point.

## Discussion

In response to caloric restriction there is an abrupt change from a state of energy balance (weight maintenance) to a negative imbalance, which eventually will reach a new equilibrium at a lower body mass when the decline in energy expenditure is maintained at a level equivalent to the energy intake. Whether the decline in energy expenditure is equal or larger, than the reduction in metabolic mass is still debated. Several reports suggest that despite weight stability, the reduction in energy expenditure can be lower than one would expect for the new metabolic weight and composition [20]–[23]. This ‘physiological adaptation’ is postulated to be an integral factor contributing to protection against excessive weight loss during caloric restriction and importantly predisposing to weight regain in post-obese individuals [21]. It is unclear if only some or all, of the components of daily energy expenditure contribute to the disproportionate decline or ‘adaptation’ in energy expenditure in response to caloric restriction. Additionally the role of exercise on the metabolic adjustments to CR interventions is not known.

**Figure 4:**
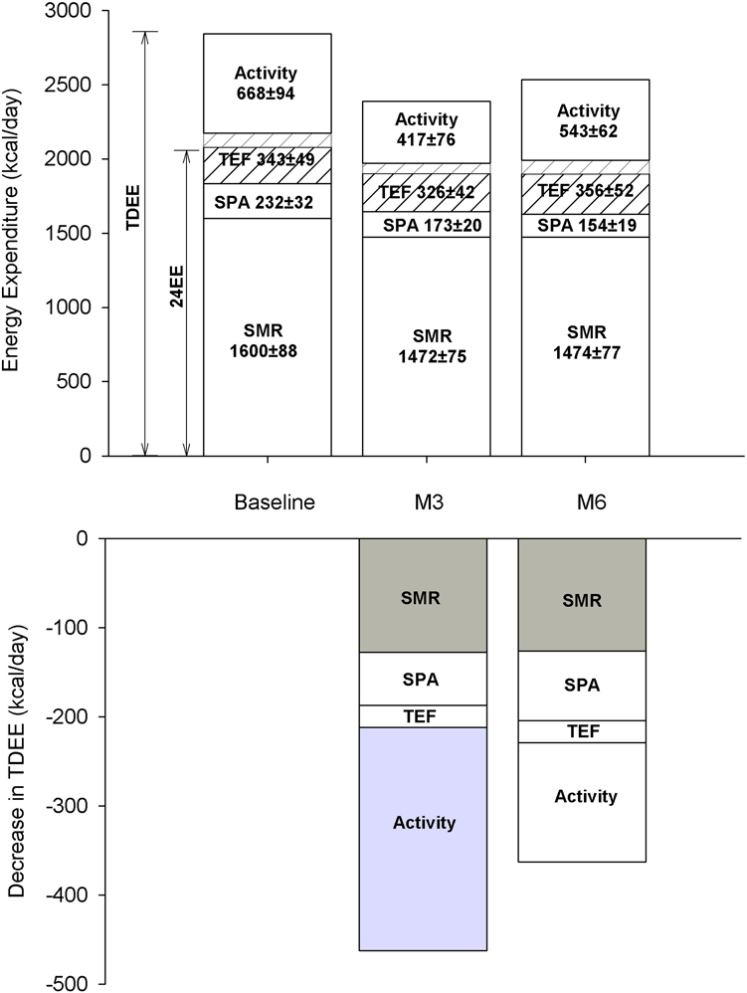
The effect of caloric restriction (CR, n = 12) on all components of daily energy expenditure (Top Panel). Total daily energy expenditure (TDEE) is measured by doubly labeled water over a 2-week period whereas sedentary 24-h energy expenditure (24h-EE) is measured in a respiratory chamber. Spontaneous physical activity was calculated as previously reported [7] whereas the thermic effect of food (TEF) was calculated in the chamber as per Tataranni et al [38] and the same percentage of energy intake (9–12% across subjects) was assumed to assess the extra TEF in free living conditions. The changes in total daily energy expenditure after 3 and 6 months of CR (Bottom Panel) are shown and those representing a metabolic adaptation (larger than due to weight loss) are highlighted in grey.

Recently we reported a reduction in sedentary energy expenditure (24h-energy expenditure; 24h-EE) with 25% CR that was 6% lower than the change expected for the loss of metabolic tissues [6]. Now for the first time, we objectively characterized the response in all the components of daily energy expenditure to caloric restriction by combining doubly labeled water and indirect calorimetry (Figure 4). In response to caloric restriction (CR/LCD) we observed a true metabolic adaptation at months 3 and 6 of the intervention. To exclude the contribution of sedentary energy expenditure (the largest component of daily energy expenditure), we adjusted TDEE for sedentary energy expenditure (24h-EE and SMR) and observed that measured TDEE was significantly less than predicted at both month 3 and month 6 of CR. Interestingly no metabolic adaptation was observed in CR+EX. Together, this data indicates that TDEE is reduced with caloric restriction and is likely the result of a metabolic adaptation in the sedentary state accompanied by a reduction in activity-related energy expenditure and reduced levels of physical activity (Figure 3). Therefore, this study supports a ‘metabolic adaptation’ in response to weight loss in humans and demonstrates for the first time a reduction in all components of daily energy expenditure with dietary-induced weight loss, including the level physical activity. Importantly, CR in combination with exercise (CR+EX) did not result in metabolic adaptation while inducing similar changes in body composition as with CR alone.

The concept of an adaptation in metabolic rate in response to caloric restriction (defined as reduction in energy expenditure that is more than would be expected on the basis of the loss of metabolic mass) was proposed by Keys et al in the 1950's [8]. In this pioneering investigation where 32 lean men were fed only 50% of the required energy intake for 24 weeks, basal oxygen consumption was decreased by 31%, 19% and 16% after adjusting for the loss of body surface area, body weight and “active tissue”, respectively. Behaviorally, a response to the semi starvation was also a tremendous decrease in physical activity. It was estimated that reduced physical activity accounted for 58% of the decreased total energy expenditure whereas RMR accounted for 32% and the thermic effect of food for only 10% [24].

Studies in obese individuals have also reported a metabolic adaptation (measured by RMR adjusted for body composition) with weight loss [25]–[27]. Furthermore, in other landmark studies, a 10% weight loss in lean subjects [21] resulted in a 10–15% lower energy requirement for weight maintenance even after adjustment for fat-free mass [21], [23]. These adaptations in metabolic rate could be explained by an improved metabolic efficiency of the skeletal muscle or as also postulated, due to a reduction in physical activity with weight loss [28].

The energy cost of physical activity is proportional to body weight. Therefore, this component of energy expenditure decreases with weight loss even in the absence of a reduction in physical activity. A decrease in spontaneous physical activity has been reported in some [29] but not all weight loss studies [9], [10], [21] including our own caloric restriction study [7]. With regard to free-living energy expenditure, two studies of CR in non-obese humans suggest that after accounting for changes in body energy stores, a reduction in TDEE was partly due to lower levels of physical activity (PAL) or improved energy efficiency of physical activity at the new body weight and body composition [30], [31]. Our data supports the concept that in addition to the metabolic adaptation, reduced energy expenditure with CR is also due in part to lower physical activity level, i.e. a “behavioral adaptation”. Consistent with our study of CR, male rhesus monkeys undergoing 30% CR had reduced physical activity in comparison to control animals after 1 year of CR [32]. Despite the observed decline in physical activity, self-assessed physical functioning by the quality of life measure (SF-36) was significantly improved in the CR group at month 3 and in all intervention groups at month 6. Physical functioning has been shown to be related to physical activity level [33] therefore the observed reduction in physical activity in response to CR maybe an unconscious phenomenon used by individuals to conserve energy during energy deficit.

Interestingly, despite similar body mass and composition changes, CR in conjunction with exercise (CR+EX) did not result in a metabolic adaptation. If weight relapse does occur in part as a result of a reduced metabolic rate in the weight reduced state, then perhaps the combination of CR and exercise may be the best choice of intervention to prevent weight regain in overweight and obese individuals. Certainly, more than 20 years ago, Pavlou observed that exercise during a CR-induced weight loss program was essential for success of weight loss maintenance [34]. Since then others have shown with doubly labeled water studies that weight stability following weight loss is sustained by higher levels of activity related energy expenditure and free-living physical activity [35], [36]. To our knowledge no studies have prospectively studied the energetic adjustments of CR only versus CR in conjunction with exercise during weight loss and weight loss maintenance.

Whether or not an individual responds to weight loss with a metabolic adaptation has long-term importance for weight maintenance because there is recent data indicating that the metabolic adjustments occurring as a result of CR and weight loss are maintained for up to 6 years following the weight loss [22]. As an alternative hypothesis it would be reasonable to assume two metabolic phases occur in response to CR; an ‘adaptive phase’ when energy expenditure is adjusting to compensate for the reduced availability of energy sources, followed by a ‘maintenance phase’ when energy balance has been re-established and the metabolic and behavioral compensations are therefore complete [37]. The individual group data for CR and LCD in our study may support this viewpoint since we observed a metabolic adaptation early (M3) but a return towards baseline values after an additional 3 months (M6) of weight loss (CR) or weight stability (LCD). We could also conclude that failure to detect a statistically significant adaptation at month 6 however, may be due to limitations in sample size. Considering our sample size of 12 in CR, and the observed mean change in TDEE at 6 months (CR group) adjusted for changes in FFM and FM (−209±114 kcal/d), we would have required 22 volunteers to detect a significant change from baseline with 80% power. This is probably why we did observe a significant metabolic adaptation at both 3 and 6 months of intervention when combining the CR and LCD groups.

In this study, we combined two state of the art methods (indirect calorimetry and doubly labeled water) for quantifying precisely the complete energy expenditure response to caloric restriction in non-obese individuals. We identified reduction in sedentary energy expenditure that was 6% larger than what could be accounted for by the loss in metabolic size [6], i.e. a ‘metabolic adaptation’. This report provides further evidence that a metabolic adaptation in response to CR can be found in the free-living situation as well. This adaptation comprises not only a reduction in cellular respiration (energy cost of maintaining cells, organs and tissue alive) but also a decrease in free-living activity thermogenesis. These observations are of importance to understand the progressive resistance to weight loss seen in so many studies in which weight plateaus after 6–12 months of caloric restriction despite self-declared adherence to a hypocaloric dietary prescription. Furthermore, our data shed some light on lifestyle change interventions that combining diet and physical activities are probably more successful in maintaining weight loss longer term.

## Supporting Information

Checklist S1 Consort checklist
(0.06 MB DOC)

Protocol S1 Study protocol
(0.36 MB DOC)
